# Properties of High-Entropy Fe_30_Co_20_Ni_20_Mn_20_Al_10_ Alloy Produced by High-Energy Ball Milling

**DOI:** 10.3390/ma17010234

**Published:** 2023-12-31

**Authors:** Chérif Ben Ammar, Nawel Khitouni, Wael Ben Mbarek, Abdulelah H. Alsulami, Joan-Josep Suñol, Mohamed Khitouni, Mahmoud Chemingui

**Affiliations:** 1 Laboratoire de Chimie Inorganique, UR 11-ES-73, Université de Sfax, B.P. 1171, Sfax 3018, Tunisia; cherif23495@gmail.com (C.B.A.); khitouninawel@yahoo.fr (N.K.); chmingui_mahmoud@yahoo.fr (M.C.); 2 Department of Physics, Campus Montilivi, University of Girona, 17071 Girona, Spain; benmbarek.wael@hotmail.fr; 3 Chemistry Department, Faculty of Science and Arts in Baljurashi, Al-Baha University, Al-Baha 65527, Saudi Arabia; aalsulami@bu.edu.sa; 4 Department of Chemistry, College of Science, Qassim University, Buraidah 51452, Saudi Arabia; kh.mohamed@qu.edu.sa

**Keywords:** HEA alloy, mechanical alloying, X-ray diffraction, microstructure, magnetic characteristics

## Abstract

A high-entropy Fe_30_Co_20_Ni_20_Mn_20_Al_10_ (at%) alloy with a face-centered cubic (FCC) crystalline phase was produced through mechanical alloying. This study examined the development of its phases, microstructure, morphology, and magnetic characteristics. Scanning electron microscopy (SEM) was applied to assess the sample morphology in relation to milling times. The changes that the material underwent during milling were investigated using X-ray diffraction. The milling time affected the phase transformation. A single FCC solid solution (crystallite size = 12 nm) was found after 50 h of milling. Additionally, the magnetic characteristics were examined and shown to be associated with microstructural changes. The powder mixture exhibited behavior consistent with soft magnetics, with an *Hc* value of 8 Am^−1^ and an *Ms* value of 165 emu/g. The excellent soft magnetic characteristic may be related to the stability of the FCC phase, which was generated following a 30 h milling process. In addition, the low value of *Ms* may have originated from the presence of Al atoms in the solid solution and the development of large densities of interfaces and crystal defects.

## 1. Introduction

Generally, bimetallic and trimetallic alloys have been effectively employed, with one of the added metals acting as the major component and being frequently used in greater quantities (solvent element) while the other additional metals (solute elements) are often employed in smaller amounts [1,2]. Nonetheless, it has been stated that remarkable progress has been made in the last several decades in the development of specific alloys, such as superalloys and stainless steel. The development of these alloys was based on the complexity of their chemical compositions [3]. Typically, they are made up of many elements and have higher mixing entropies than pure metals. However, it is possible to add more alloying elements because as the alloy mixing entropy rises, so does its mixing enthalpy, and the alloy characteristics are consequently markedly enhanced. The new metal alloys are known as high-entropy alloys (HEAs). More than a decade ago, HEAs were first introduced as a novel class of multicomponent alloys by Yeh and co-authors. [4] and Cantor et al. [5]. HEAs frequently form a duplex (FCC + BCC)- or a single FCC- or BCC-solid solution [6]. Amorphous structures can be seen in some other HEAs [7]. Because of their exceptional mechanical characteristics, such as hardness and resistance to wearing, as well as their soft magnetic qualities, HEAs are thought to be very promising materials [8]. HEAs have been produced via powder metallurgical processes, deposition techniques, and melting and casting methods [9]. The large compositional space as well as complex dimensions of HEAs are further enhanced by the addition of a nanocrystalline structure. Furthermore, it has been reported that nanostructured HEAs have good magnetic behavior [10], greater thermal stability [11], and superior mechanical properties [12]. The mechanical alloying (MA) method is a popular approach for creating nanoscale solid solution structures with unique characteristics and provides a substitute for arc melting and the use of foundries for producing high entropy alloys [13,14]. MA provides the benefit of extended solid solubility even in immiscible solutions. This might be explained by the higher diffusion times brought on by the nanosize of the powder components before the alloying process. As a result, MA gives phases in HEAs greater stability in addition to increasing configurational entropy. A detailed analysis of the impact of various milling factors on the composition and behavior of HEAs was carried out by Murty et al. [15]. These factors include the kind of milling device, the atmosphere, the ball-to-powder ratio, the milling frequency, and the processing time. Indeed, by modifying milling parameters including the milling time, milling frequency, process control agents, milling media (atmosphere, dry or wet milling), and ball-to-powder ratio, there is an opportunity to increase the energy efficiency in the bulk manufacturing of HEAs. Moreover, the extension of the solid solution and the creation of nanocrystalline/amorphous materials can result from the production of a high degree of crystallographic defects, or grain boundaries, by MA and the segregation of solutes at these defects [16]. High-energy mechanical milling was used by Varalakshmi et al. [17] to create the first HEA, AlCrCuFeTiZn with a BCC structure and crystalline size of about 10 nm. They reported that after annealing the alloys for 60 min at a temperature of 800 °C, they remained stable. Moreover, Gómez-Esparza et al. [18] synthesized the HEA FeCoNiAlCr using MA and revealed that the powder exhibited a mixture of the cubic BCC- and FCC-phases following ten hours of milling. In a relatively recent work, the HEA FeCoNiB_0.5_Si_0.5_ was properly formed using MA for 150 h with a single solid solution phase; the thermodynamic criteria for creating such a phase were not identified [6]. On the other hand, recent studies have revealed that HEAs exhibit excellent soft magnetic characteristics in addition to their extraordinary mechanical properties [19,20]. Based on the work of Zuo et al. [19], the as-cast magnetic HEA CoFeMnNi has an inferior *Ms* value of 18.14 emu/g, whereas the as-cast AlCoFeMnNi alloy has an elevated *Ms* value of approximately 148.7 emu/g. As a result, the addition of Al considerably increased its saturation magnetization. To meet the increasing performance and technological requirements in the field of electronics, a unique soft magnetic material should be developed. New opportunities for developing innovative soft magnetic materials have been made possible by the introduction of HEAs [10,19,20]. HEAs developed from ferromagnetic materials (Fe, Co, and Ni) often also have good soft magnetic properties. Transformers and motors, two types of electromagnetic equipment utilized in the electronics industry, depend on these soft magnetic materials. A good soft magnetic material must have low coercivity, electrical resistivity, and magnetic permeability and high saturation magnetization. For instance, the CoFeMnNiGa [20] and CoFeMn_0.25_NiAl_0.25_ [19] alloys exhibit low coercivity and high saturation magnetization. Additionally, recent measurements of magnetization when Al is added to the HEA FeCoNiCr have demonstrated that, at room temperature, the magnetic state changes gradually from paramagnetic to ferromagnetic [21]. The influence of an additional component on phase formation and alloy characteristics, however, depends on several variables, including the radii of atoms, the crystal structure, and the mixing enthalpy with other elements in the alloy. For example, the phase evolution in HEAs is most significantly influenced by Al, and through sequential alloying, Vaidya et al. [22] showed how Al can promote the creation of the BCC phase. Additionally, due to its considerable ductility, it results in lower contamination levels because it erodes milling media less frequently. Additionally, in a soft CoCrFeMnNi-based FCC alloy, Al addition causes ordered B2 precipitates to form and harden [23]. According to the previously mentioned studies, HEAs have advanced significantly in a range of functional applications, including superconducting materials, diffusion barrier films, photothermal conversion materials, soft-magnetic materials, corrosion resistance materials, and irradiation resistance materials. Since there are several gaps in our understanding of the relationship between the structures, microstructures, and functional properties of these materials, it is essential to research functionally oriented HEAs. Consequently, this work focuses on the synthesis and analysis of the effect of the milling time on the HEA FeCoNiMnAl during the mechanical alloying procedure. The structural, microstructural, and magnetic characteristics are examined for this purpose.

## 2. Materials and Methods

A high-energy planetary laboratory mill device (Fritsch Pulverisette P7, FRITSH GmbH, Idar-Oberstein, Germany) was employed to mechanically mill the elemental powders of Fe, Co, Ni, Mn, and Al (purity~99.5%; particle size ≤ 30 µm; from Alpha Aesar, Haverhill, MA, USA) in an argon atmosphere in order to achieve the desired composition of Fe_30_Co_20_Ni_20_Mn_20_Al_10_ (at%). Experiments using mechanical alloying were conducted in a stainless-steel container that had been hardened. The powder-to-ball weight ratio was kept constant at 0.47. A 600 rpm milling speed adjustment was made. The processing favors the formation of powder agglomerates, and adhesion to the container walls and balls were avoided using a milling sequence that involved 10 min of milling followed by 5 min of idle time. 

Using scanning electron microscopy (SEM, DSM960A ZEISS, Carl Zeiss GmbH, Oberkochen, Germany) in secondary electron mode at a voltage of 15 kV, the morphology of the particle powders was examined. The energy dispersive X-ray spectrometry (EDS) analyzer Vega©Tescan (Brno, Czech Republic) was mounted in the SEM. The program Image J (version 1.x) was used to determine the powders’ particle sizes.

The milled powders were examined via X-ray diffraction (XRD) using a Siemens D500 powder diffractometer (Berlin, Germany) in (θ–2θ) geometry using CuKα radiation (λ_Cu_ = 0.15418 nm). The phase analysis was performed using ICDD (PDF-2, 2012) files. Both structural and microstructural parameters were determined from the refined products’ XRD patterns using the MAUD program (version 2.55) based on the Rietveld method [24]. 

A thorough analysis of the XRD profiles can be carried out in the framework of MAUD software (version 2.9) [24] using the Rietveld refinement and Warren–Averbach methods [25,26]. This will enable the determination of the phase compositions as well as the structural and microstructural parameters for each phase, including the lattice parameters, average crystallite size ⟨D⟩, root-square lattice strain ⟨ε^2^⟩^1/2^, and stacking fault probabilities (SFP).

Rietveld refinement was carried out in MAUD (version 2.55) software. It automatically carries out the best-fit refinement using the databases and the trial patterns [27]. This interesting program is available for free at (http://maud.radiographema.com/) (accessed on 22 May 2023) [28]. MAUD requires two files: the network parameters of the phases that need to be refined (*. CIF format) and the patterns to be refined (*.dat or *.xy formats). The following strategies were used when employing the MAUD program: (i) The preparation of experimental XRD patterns of studied samples, with verification of the quality of this data; (ii) A qualitative analysis (XRD and XRF dual analysis) to determine the phases found in the sample; (iii) The loading of standard data (*.CIF) for each phase (it describes the phase information: symmetry, locations of atoms, etc). The latter data can be obtained from ICSD databases or the COD (Crystallography Open Database); (iv) Starting the MAUD program, which contains a graphical user interface (GUI); (v) Entering experimental XRD patterns; (vi) Entering the *.CIF files of phases composing the sample; (vii) Establishing certain parameters: background, 2θ range (θ_min_, θ_max_), etc.; (viii) Commencing standard refinement: refinement of the background and density (a match must be made between the experimental and calculated peaks); (ix) Refinement of atomic locations, shapes, and structures of peaks (by improving the crystallite size and microstrain parameters); (x) Launching the quantitative analysis command. 

It is feasible to compute a statistical parameter, known as the goodness of fit “*χ*^2^”, which is the ratio of *R_wp_* to *R_exp_* and which must increase towards unity for a successful refinement:

$$\chi^{2}=\frac{R_{wp}}{R_{exp}}\;$$

The profile refinements continue until convergence is reached; the value of the quality factor *χ*^2^ (GOF) approaches 1.

The SQUID MPMS-XL superconducting quantum interference equipment of Quantum Design (Caledonia, MI, USA) was employed to measure the saturation magnetization (*Ms*), remanence (*Mr*), and coercive field (*Hc*) of the as-alloyed powders at 300 K with a maximum applied field of 50 kOe.

## 3. Results

### 3.1. SEM Analysis

**Figure 1:**
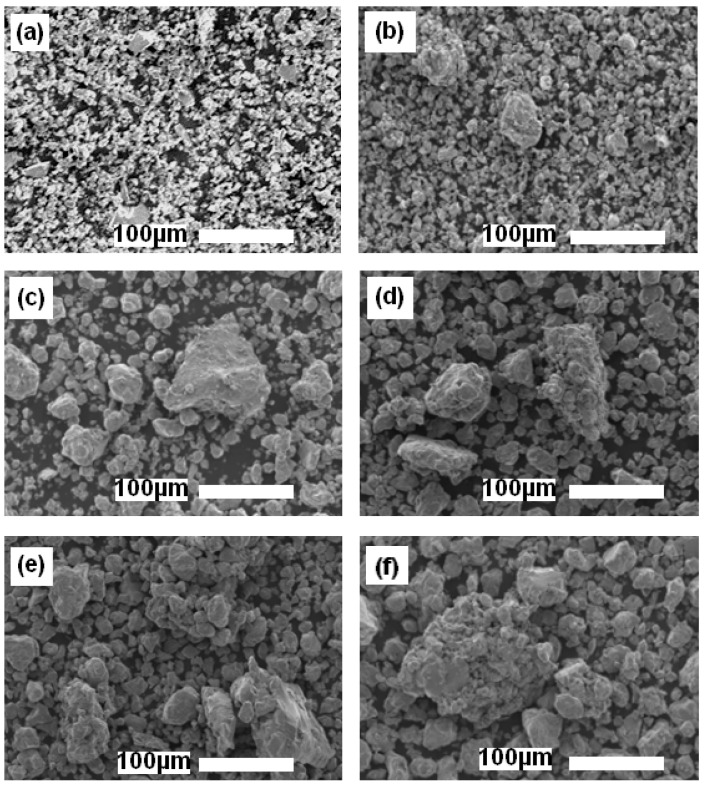
SEM photos of the MA Fe_30_Co_20_Ni_20_Mn_20_Al_10_ powder mixture obtained after various MA times: (**a**) 0 h, (**b**) 4 h, (**c**) 10 h, (**d**) 20 h, (**e**) 50 h, and (**f**) 100 h.

SEM micrographs of the alloyed Fe_30_Co_20_Ni_20_Mn_20_Al_10_ (at%) powder mixtures produced before (Figure 1a) and after high-energy mechanical milling for 4, 10, 20, 50, and 100 h (Figure 1b–f) are shown in Figure 1. As shown, each image was obtained at a 100 µm scale length using an ×300 magnification. The majority of the particles were smaller than 30 µm, and cold-welded particle clusters with sizes up to 150 µm were formed. 

The particle shapes ranged from spherical to polygonal. The final microstructure was the result of two deformation mechanisms, fracture in hard powders and plastic deformation linked to cold welding in ductile powders, as previously reported [29,30]. Notably, Al particles are the softest and most ductile of all the metal mixtures; they were therefore severely deformed and could bond the hardest particles into large cold-welded particles (Figure 1c–f). However, the state of the tiny particles may be the result of the intensive fracturing of particles (Figure 1b). This is due to the dissolution of metallic elements which form supersaturated solid solutions and an increase in hardening (longer milling times resulting in a greater percentage of crystallographic imperfections). 

### 3.2. XRD Analysis

**Figure 2:**
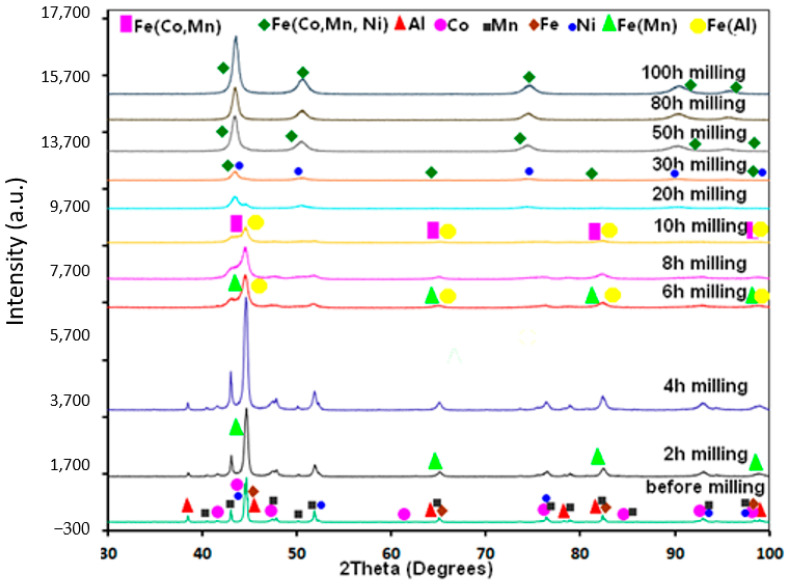
XRD patterns of the MA Fe_30_Co_20_Ni_20_Mn_20_Al_10_ powdered specimens collected after selected MA times.

**Figure 3:**
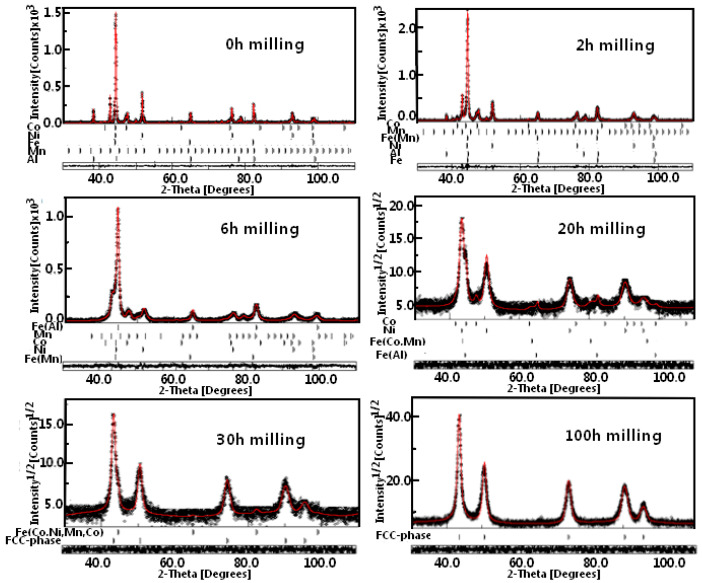
X-ray diffraction experimental patterns and the associated Rietveld theoretically adjusted MA of Fe_30_Co_20_Ni_20_Mn_20_Al_10_ powders collected after selected milling times. The symbol x in x10^3^ is a multiplication sign.

Figure 2 displays the X-ray diffraction patterns of Fe_30_Co_20_Ni_20_Mn_20_Al_10_ powder mixtures as a function of milling time. Figure 3 also displays the Rietveld investigations of the XRD patterns. There was always a refinement parameter for goodness of fit (GOF) less than 1.12. Controlling the alloying process was made possible by the subsequent diffraction patterns.

The X-ray pattern of the powder before milling is shown for comparison. The BCC Fe (Im-3 m; a = 2.8667(1) Å), HCP Co (P63/mmc; a = 2.5071(1) Å and c = 4. 0713(1) Å), FCC Ni (Fm-3 m; a = 3.5260(1) Å), BCC Mn (I-43 m; a = 8.9125(1) Å), and FCC Al (Fm-3 m; a= 4.0478(4) Å) are the peaks that were recorded before milling (Figure 2 and Figure 3). There was a gradual disappearance of the Al peaks located at 2θ = 38.48° during milling, which suggests that the MA caused the Al to dissolve in the other metal’s lattice. After 6 h, this peak completely vanished, showing that Al was introduced into the BCC Fe lattice to form the supersaturated solid solution BCC Fe(Al) (Im-3 m; a = 2.8668(2) Å, wt% = 20%) (Figure 2 and Figure 3). In contrast, a disordered BCC Fe(Mn) solid solution (Im-3 m; a= 2.8670(1) Å, wt%~11%) appeared after the first 2 h of milling. This phase was also refined after 6 h with a lattice parameter of 2.8637(12) Å and a phase proportion of 40.891%. We found that the BCC Fe(Co,Mn) supersaturated solid solution with a lattice parameter of 2.9147(1) Å and a phase proportion of approximately 55% appears when the milling period is extended to 20 h. At the same time, we refined the BCC Fe(Al) phase (a= 2.8662(1) Å; wt% = 14.4) with a proportion of HCP Co (a = 2.5071(1) Å and c = 4. 0713(1) Å; wt% = 14.6) and FCC Ni (a = 3.6057(1) Å; wt% = 16) phases. With milling time, the (110) BCC Fe strongest diffraction peak became asymmetrical and shifted toward smaller angles, which suggests that the lattice parameter increased. After 30 h of milling, the BCC and FCC solid solutions converged, which can be attributed to the diffusion of Co, Ni, Mn, and Al into the BCC Fe matrix, which caused the lattice expansion (see Figure 3).

An earlier report [31] stated that a new FCC structure with a lattice parameter greater than the FCC Ni phase is formed as a result of the interdiffusion of Ni and Fe. The BCC iron peak disappears as the milling time exceeds 30 h, leaving only the peaks associated with an FCC solid solution phase apparent. Furthermore, the BCC phase disappears after 50 h, as shown by the significant displacement of the peak to the lower angle (see Figure 3). Consequently, this refined FCC phase at extended milling times is a result of the diffusion in the Ni matrix. Rietveld refinement using the FCC FeCoMnNiAl phase (a = 3.6250(1) Å, wt% = 100) was acceptable after 100 h of milling (Figure 2 and Figure 3). A considerable amount of enthalpy could be preserved in nanocrystal alloys due to the large grain boundary domain and the large number of defects that occurred during grain polishing with nanometric crystallites [32]. As a result, the energy contained in the crystalline lattice’s distortion and grain boundaries can help a solid solution form rapidly. Furthermore, the lattice may deform due to the surface tension of nanometric grains, increasing the solubility. Chen et al. [33] reported an inverse correlation between the melting points and alloying efficiencies for elements with similar concentrations. The element’s diffusivity in the solid-state increases with decreasing melting points [34]. Elements with low melting temperatures, such as Fe (1811 K), Co (1768 K), Ni (1726 K), and Mn (1519 K), have alloying rates that are directly correlated with the softness of the pure element [33]. Furthermore, as the element concentration decreases, the element’s alloying rate rises [35]. As a result, the elements in the FeCoNiMnAl HEA should diffuse in the following order: Al → Mn → Co → Ni → Fe.

**Figure 4:**
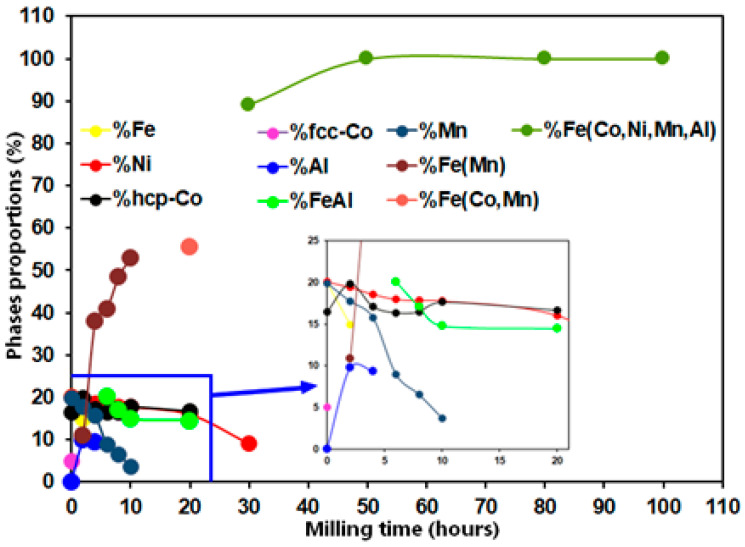
Variation in phase proportions as a function of milling time.

**Figure 5:**
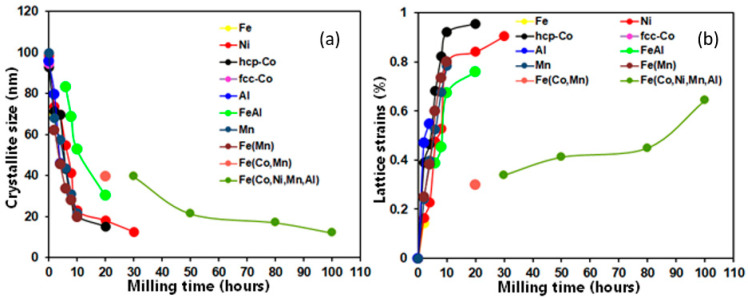
Variation in crystallite sizes (**a**) and lattice microstrain values (**b**) versus MA time.

The phase proportion evolution with the milling time is calculated by refining XRD diffraction patterns with the MAUD software. The results are shown in the Figure 4. Likewise, the change in the lattice strains and estimated crystallite sizes versus the MA time is displayed in Figure 5. It is apparent that when milling duration increases over 20 h, the grain sizes gradually reduce. The estimated values after 20 h of milling were approximately 15, 18, 30, and 39 nm for the FCC Ni, HCP Co, BCC Fe(Al), and Fe(Co,Mn), respectively (Figure 5a). Then, the size of the crystallite of the FCC Ni phase continued decreasing up to 12.5 nm. For higher milling times, the FCC FeCoNiMnAl phase is formed with a size that is 39.41 nm greater than that of Ni after 20 h milling because of the effect of the solid solution and the dynamic recrystallization of grains created due to local heating during milling [36,37]. Then, at the end of the milling step, the size drops to a value of 12 nm. In parallel, the lattice strain levels for all elements increase steadily over the first 20 h of milling, reaching values of 0.84, 0.95, 0.76, and 0.3% for the FCC Ni, HCP Co, BCC Fe(Al), and Fe(Co,Mn), respectively (Figure 5b). As the final byproduct of the MA process, the value of the lattice strains of the FCC FeCoNiMnAl phase slowly increases from 0.337% (after 30 h of milling) to 0.64% at the end of milling (Figure 5b). Lattice strain increases are often caused by significant dislocation densities and an increase in the grain boundary proportion [38]. Dislocations represent a significant concern during the refining process. In particular, the early stages of milling are characterized by the generation of dislocation cell blocks, separated by dense dislocation walls and containing dislocations arranged cellularly within them. The inner dislocation structure becomes more random and has a smaller space for cellular structures as the strain grows. Additionally, the density of heavy dislocation walls increases, and the size of the cell blocks becomes closer to that of a cell when the strain lowers.

### 3.3. Magnetic Analysis

**Figure 6:**
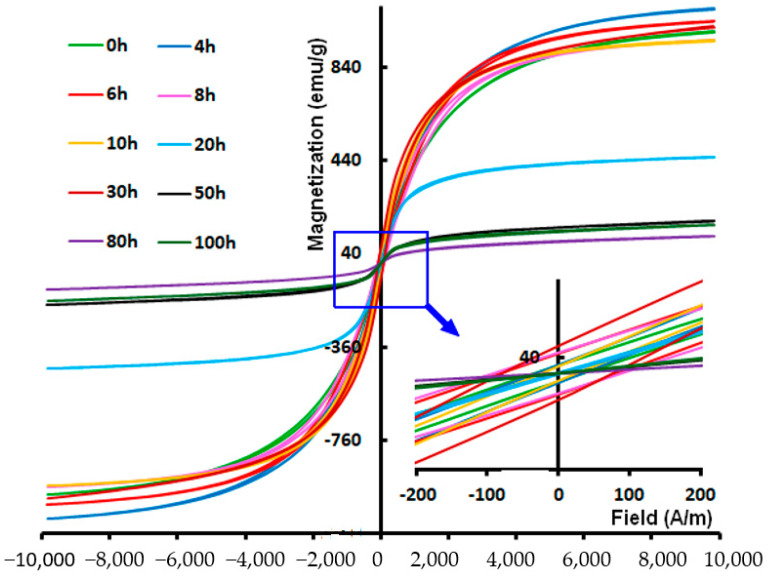
Hysteresis cycles (at 300 K) of the milled Fe_30_Co_20_Ni_20_Mn_20_Al_10_ powder as a function of milling times.

Figure 6 displays a superposition of the hysteresis loops (M–H) of the MA Fe_30_Co_20_Ni_20_Mn_20_Al_10_ powder mixtures as a function of the selected milling time. Similar hysteresis loops were present in all of the milled powders, showing that these samples exhibit ferromagnetic activity. Furthermore, every hysteresis cycle displayed a sigmoidal form with minimal loss, which is indicative of behavior found in nanostructured materials with tiny magnetic domains [39]. Furthermore, soft magnetic materials are required to have extremely low hysteresis losses [40].

**Figure 7:**
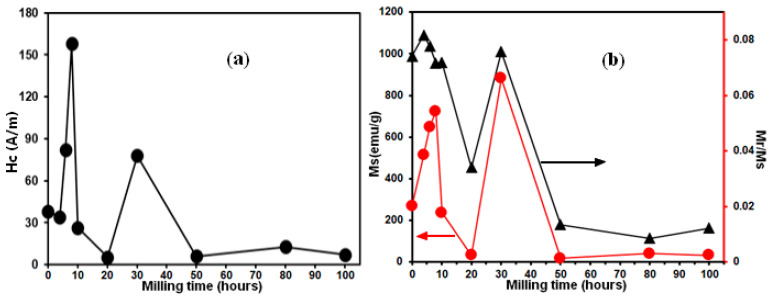
(**a**) Variation in the coercivity (*Hc*) and (**b**) magnetization of saturation (*Ms*) and the *Mr*/*Ms* ratio versus MA time.

The correlation between the magnetization of saturation (*Ms*) and coercivity (*Hc*) as a function of MA time is displayed in Figure 7. In general, the hard magnetic materials have an *Hc* of up to 2800 kA m^−1^ (35.18 Oe), while the majority of the soft magnetic materials have an *Hc* of less than 1000 Am^−1^ (12.56 Oe) [41]. As shown in Figure 7a, the magnetic behavior of all mechanically alloyed powders is soft (*Hc*~158 Am^−1^ (~2Oe)). Coercivity increases quickly during the initial 8 h of the milling procedure, reaching 158 Am^−1^. According to reports, the increase in *Hc* values corresponds to a decreased coupling between ferromagnetic grains via the intergranular region [42,43]. Moreover, due to the ferromagnetic nature of the Fe, Co, Ni, and Mn metals, substituting one of these elements with the non-magnetic Al results in a reduction in ferromagnetic coupling [44]. On the other hand, dislocation density has also been identified as another crucial element influencing coercivity [45]. In the same context, Yu et al. reported experimental results demonstrating a direct relationship between an increase in *Hc* and the appearance of grain boundaries, precipitation, and disorderly processes [46]. Surface anisotropy could be another factor contributing to enhanced *Hc* [47,48,49]. The latter phenomenon could have its origin in the exchange connections between the spins of the surface and core atoms [47,48,49,50]. Surface anisotropy becomes more significant as the particle size is reduced to the nanometric interval because of the increased surface/volume ratio [51,52]. Longer milling times cause *Hc* to decrease to 5.4 Am^−1^. This reduction can be linked to the slow variation in grain size and microstrain as well as the decline in magnetocrystalline anisotropy. After 30 h of milling, *Hc* rises once more to a value of 78 Am^−1^ before decreasing once again to around 8 Am^−1^ and remaining constant up to the end of milling. 

It is well known that it is possible to characterize the coercivity, or *Hc*, variations during the milling process as a progressive rise dependent on the milling time. Coercivity behavior can be impacted by both the introduction of different structural faults and the refining of grain size during the milling process. Therefore, the increase in coercivity may be attributed to the fact that the grain sizes are larger than the thickness of the domain wall and that the grain boundaries serve as barriers to domain wall motion. According to the random anisotropy model [53], when the ferromagnetic exchange length, Lex, is larger than or equal to the grain size, *D*, and the inverse grain size as a function of milling time, then *Hc* obeys a 1/*D*-dependence law. As previously reported [54], the coercivity specified by grain boundaries can be written as follows:

$$Hc\sim 3\frac{\gamma_{\omega }}{Ms}\frac{1}{D}$$

where $\gamma_{\omega }\;$is the wall energy and Ms is the saturation magnetization. The wall energy, $\gamma_{\omega },\;$can be estimated using the equation:

$$\gamma_{\omega }\sim \sqrt[{3}]{\frac{k_{B}T_{c}K_{1}}{a}}$$

thus:

$$Hc\sim \sqrt[{3}]{\frac{k_{B}T_{c}K_{1}}{aMs}}\;\frac{1}{D}$$

where $k_{B}$ is the Boltzmann constant, $K_{1}$ is the magneto-crystalline anisotropy, $T_{c}\;$is the Curie temperature, and a is the lattice constant. 

Lighter information on the mechanics behind mechanical alloying was provided by the fluctuations in saturation magnetization (*Ms*) with milling time. This magnetic property can be computed using the curve (*M–H*), which represents the atomic structure of magnetism. The saturation magnetization can therefore be explained by the electronic structure, magnetic exchange between its dipoles, and the chemical composition of the alloy. However, each of these properties is greatly influenced by the quantum processes that exist and the local surroundings of the atoms [55]. Furthermore, the *Ms* was determined through the subsequent application of the classical approach to saturation [56]:

$$M=Ms\left(1-\frac{a}{H}-\frac{b}{H^{2}}\right)-\chi H$$

where *H* is the applied field, χ is the field-independent susceptibility, and a and b are coefficients that depend on the magnetic and structural properties of the sample [57,58]. According to semi-empirical relationships [59,60], the following relationship is generated under the assumption of a random exchange interaction and the application of a field high enough to fully saturate the sample:

$$A=\alpha \left(4\rho \pi Ms\right)P_{eff}$$

where α is a constant, which is usually around 0.1, ρ is the density of the material, and $P_{eff}$ is the effective fraction of porosity and non-magnetic inclusions [59,60].

Rather than displaying an evolution based on processing time, as shown in Figure 7b, the *Ms* is mostly dependent on the evolution of the system composition. However, within the initial four hours, the *Ms* value increased slightly from 989 emu/g to 1091 emu/g. After 20 h, it dropped to 455 emu/g, and after 30 h, it rose once again to 1011 emu/g. After 30 h, the observed condition reaches *Ms* at approximately 165 emu/g. The crystal structure and the quantity of magnetic components contribute to these changes in the *Ms* parameter. Since the crystal structure provides the orientation arrangement of the magnetic moment with a geometric basis, the composition and crystal structure can strongly influence the saturation magnetization [61,62].

As the milling process continues, the proportion of magnetic crystalline phases diminishes, causing the magnetism to dissipate and limiting crystallinity [12]. The coexistence of magnetic phases, namely, FCC and BCC phases, leads to a greater *Ms* in the sample milled for 30 h, as reported previously [7,61]. However, extended milling durations cause the orientational magnetic moment in the crystalline magnetic phases to be destroyed, which lowers *Ms*. [62]. The variation in *Ms* is frequently linked to a change in the proportion of the magnetic phases comprising the magnetic components Fe, Ni, and Co, which are present in the solid solution phases. The saturation magnetization increases with the proportion of magnetic phases because it increases the total magnetic moment per unit of mass [61,63]. The resultant enrichment of the solid solution Fe(Co,Mn,Ni) phase in the formed alloy at 30 h subsequently generates the increase in saturation magnetization. Furthermore, the primary cause of the long-term mechanical alloying’s decrease in *Ms* is the creation of high densities of defects and interfaces, which significantly restrict the ability of domain walls to move [64,65]. The interaction between the ferromagnetic Fe, Co, and Mn atoms and the non-ferromagnetic Al atoms is another factor contributing to the drop in *Ms*. Indeed, the Al atoms diminish the magnetic moment of the individual sites of Fe, Co, and Mn because they mediate an anti-ferromagnetic super-exchange interaction between the ferromagnetic atoms and decrease the direct connection between ferromagnetic M–M sites [66,67]. According to Plascak et al. [68], Al decreases the magnetic moment of individual Fe sites by reducing the direct ferromagnetic interaction between Fe–Fe sites as well as by mediating an anti-ferromagnetic super-exchange interaction between Fe sites through Al atoms. Further, Sato et al. [69] discussed the reason why the Al addition improved the soft magnetic properties and reported that there are two major factors influencing the soft magnetic properties: (i) the microstructure and (ii) the intrinsic properties of the material. They indicate that the Al addition to Fe generally decreases the crystal magnetic anisotropy (K_u_) and reduces the magnetostriction constant (λ). It is believed that it is reasonable that such a change in the coercivity is a function of milling time where Al diffusion progresses, and it influenced the soft magnetic properties shown in Figure 7a. Furthermore, through their simulation results of the addition of Al to the FeCoNiMn alloy, Feng et al. [70] presented another term in the enhancement of the soft magnetic character of alloys: the short-range order (SRO) parameter. According to their findings, the SRO dramatically alters the atomic nearest-neighbor environment, which affects the alloys’ magnetic characteristics. The rise in Mn and Fe magnetic moments is primarily responsible for the enhancement of saturation magnetization. Between pairs of Al–Al, Co–Co, and Co–Ni, the SRO parameters are positive; however, between pairs of Ni–Al, Co–Al, Co–Mn, and Mn–Ni, they are negative [70].

When milling periods exceed 30 h, we see a decrease in *Ms* toward lower values in the 179–163 emu/g range. This decrease can be linked to the FCC phase’s stability [70]. They reported that Al atoms that are non-magnetic change the local atomic magnetic moments and decrease saturation magnetization. Preferentially, FeCoNiMn alloy forms FCC energetically to prevent magnetic frustration. Furthermore, during the MA process, the evolution mechanism of particle powders such as repetitive cold welding and fracturing generate distortions that eventually result in a decrease in *Ms*. On the other hand, the range of values for the (*Mr*/*Ms*) ratio in all samples processed by MA is between 0.066 and 0.003. Nevertheless, it has been noted that in single-domain particles (uniaxially anisotropic) the reduced remanence is on the order of 0.5 [53]. This indicates that the magnetic nanograins retain a multi-domain microstructure even if the particle sizes are in the nanometer range. 

## 4. Conclusions

Mechanical alloying was used to produce an HEA Fe30Co20Ni20Mn20Al10 (at%) alloy with an FCC crystal structure. Phase evolution, microstructure, morphology, and magnetic characteristics were all studied. The SEM analysis shows that plastic deformation, fracture, and cold welding are the three deformation mechanisms that interact to define the final microstructure. Due to their extreme ductility and softness, Al particles may undergo severe deformation and serve as a bonding agent to fuse the toughest particles into larger cold-welded particles. On the other hand, intensely fractured hard particles could be the result of metallic components dissolving to create supersaturated solid solutions, which would raise the work difficulty. Through the use of XRD analysis, a single FCC phase with a nanocrystallite size of 12 nm was found at the end of milling. Al has a major impact on phase formation because, as milling times increase, it progressively dissolves in the metal lattices and may eventually impose its FCC structure. Magnetic responses were investigated and were shown to be connected to the microstructural alterations. An *Hc* value of 8 Am-1 and an *Ms* value of 165 emu/g were found in the final powder mixture, which demonstrated behavior compatible with soft magnetics. This soft magnetic property could be attributed to the non-magnetic Al atoms which reduce the saturation magnetization and change the local atomic magnetic moments. Additionally, the development of large densities of structure defects can be identified as another crucial element influencing soft magnetic behavior. In industrial applications, the present HEA FeCoNiMnAl may be an excellent option if its milled powders are processed using the spark plasma sintering method.

## Data Availability

The raw data will be made available on reasonable request.
